# Acute paediatric inpatient care of children and young people admitted with self-harm or eating disorders: A single centre evaluation

**DOI:** 10.1177/13674935221107962

**Published:** 2022-06-15

**Authors:** Joseph C Manning, Tim Carter, Iszara Blake, Verity Bingham, Joanne Cooper, Andrew Turner, Takawira C Marufu, Damian Wood, Jane Coad

**Affiliations:** 1 Nottingham Children’s Hospital, 9820 Nottingham University Hospitals NHS Trust, Nottingham, UK; 2 Centre for Children and Young People Health Research (CYPHR), School of Health Sciences, 6123 University of Nottinghan, Nottingham, UK; 3 Institute of Care Excellence, 9820 Nottingham University Hospitals NHS Trust, Nottingham, UK; 4 Faculty Research Centre for Intelligent Healthcare, 2706 Coventry University, UK; 5 University Hospital Coventry and Warwickshire, NHS Trust, Nottingham, UK

**Keywords:** child, mental health, Self-Injurious Behaviour, feeding and eating disorders, Inpatients, parents, Outcome Assessment, Health Care

## Abstract

In the United Kingdom, the prevalence of children and young people (CYP) accessing acute paediatric inpatient care with mental health problems is increasing, with self-harm and eating disorders particularly prevalent. This study evaluated CYP experiences of being in receipt of acute paediatric inpatient care following either self-harm or crisis stemming from an eating disorder to inform domains for a person-centred outcome measure (PCOM). A series of stakeholder engagement events were conducted between April and July 2015 where creative approaches were used to explore stakeholder experiences of care and to identify outcome domains that were subsequently prioritised using a Nominal Group Technique. Data were analysed using inductive thematic approach, with significance scores calculated for domain statements. Ninety-six stakeholders (15 CYP, eight parents and carers, and 73 professionals) participated. Findings showed five priority PCOM domains: privacy and surveillance; holistic care; making choices, appropriate communication; working together to achieve care goals; and respect and empowerment. This single centre evaluation highlights the need for a PCOM to be developed for this patient group that comprehensively reflects stakeholders’ expectations in order to inform improvements to quality of acute paediatric care.

## Introduction

Improving children and young peoples’ (CYP) mental health and wellbeing is a global priority for healthcare professionals and policy makers (Mei et al., 2020; Patel et al., 2016). The World Health Organization report that 16% of the global burden of disease in CYP aged 10–19 years is attributable to mental health conditions (World Health Organization, 2020).

In the United Kingdom (UK), it is estimated that one in eight CYP have a diagnosable mental health condition, a substantial increase from previous years (Sadler et al., 2018). Concurrently there is an increasing prevalence of CYP accessing acute paediatric inpatient care with mental health problems, particularly presentations of self-harm and eating disorders (Hawton et al., 2012; Morgan et al., 2017; Geulayov et al., 2018; Fitzgerald et al., 2020). The initial period of acute inpatient care for these patient groups involves a range of assessments and interventions in order to ensure safety (Ougrin et al., 2012) and to address their immediate physical needs, and relevant referrals to specialist care (National Institute for Health and Clinical Excellence and National Collaborating Centre for Mental Health, 2004; Burton, 2019). Timely and relevant understanding of experiences and outcomes of those in receipt of care is pivotal in informing and developing quality services based on shared decision making and family-centred care (World Health Organization, 2016). However, for CYP accessing acute paediatric inpatient care with mental health problems there is a paucity of experience and outcome data.

The UK policy document, *No Health Without Mental Health* strategy (Department of Health (DoH), 2011) and *Future in mind* (Department for Health, 2013) identifies a shift in emphasis required to place mental health outcomes firmly alongside physical health outcomes. However, understanding all stakeholders’ (including children and young people, parents and carers, and professionals) expectations is essential to ensure delivery of cost-effective and high quality care (World Health Organization, 2016). This may in turn lead to improved recovery and quality of life (Weldring & Smith, 2013). Despite generic outcome measures available to assess quality of life and recovery for this patient group, there is currently no outcome measure that assesses quality and impact of care for CYP experiencing mental health crisis and admitted to paediatric inpatient settings.

Person reported and centred outcome measures are becoming increasingly popular within health care and can be used to measure several domains including patient satisfaction and experience of the process of care (Ovretveit et al., 2017). Centrally, such outcome measures emphasise outcomes that are important from a patient’s point of view and are often used to improve understandings between these outcomes and care provided (McGrail et al., 2011). Due to the relatively recent introduction of PCOMs in routine clinical healthcare practice, there is no universal approach to their development. However, this process may consist of a preliminary assessment of priority areas through literature searching and stakeholder (i.e. specific patient group and appropriate family/carers) involvement through an iterative process of interviews, surveys or other creative methods. Once a final set of domains are established they will typically be piloted and recipients’ interviewed to further refine the PCOM as appropriate (see, for example, Tuersley et al., 2018).

## Aim

To evaluate the experiences of CYP with self-harm or eating disorders, their parents and carers, and professionals, in order to develop the domains for a person-centred outcome measure (PCOM) that assesses quality and impact of non-specialised, acute paediatric care.

## Methods

This study was classified as a service evaluation and in accordance with UK standards meant that formal ethical approvals were not required (confirmed by: http://www.hra-decisiontools.org.uk/research/). It was registered with the governance department at Nottingham University Hospitals NHS Trust [Registration Number: 21-472C] and was conducted in compliance with the ethical principles in the Declaration of Helsinki (World Medical Association, 1996); principles of Good Clinical Practice, and UK policy framework for health and social care research (Health Research Authority, 2017). To protect the anonymity of the data source (e.g. stakeholder), pseudonyms have been used in the reporting of the findings.

### Participant recruitment and eligibility criteria

CYP, parents and carers were identified through retrospective screening of a local hospital admission database and invited via postal mail. Eligibility criteria were developed and agreed (detailed in Table 1). Due to the diverse nature of stakeholder’ characteristics such as age, ability, developmental level and professional background, methods for eliciting experiences and feedback of care were selected pragmatically and according to whether the methods were deemed suitably engaging and sensitive to the population group.

**Table 1.** Stakeholder eligibility criteria.

CYP stakeholders
- Aged 10–18 years
- Admitted to NHS trust with either eating disorder or self-harm
- 6–24 months post admission
- Not current inpatient
- Not deemed as at risk by clinician
Parents/carer stakeholders
- Parent or carer of a CYP aged 10–18 years
- Their CYP admitted to hospital in past 6–12 months with eating disorder or self-harm injury
Professional stakeholders
Including but not limited to:
- Healthcare professionals
- Social care professionals
- Education professional

Professional stakeholders were recruited from local health, social care, education and third sector organisations. They were invited directly, via email and indirectly via chain referral from first contact stakeholders.

Written informed consent was provided by every stakeholder before study activity commencement, following verbal and written explanation of the nature and purpose of the study. For stakeholders aged under 16 years, written consent was gained from the parent or legal guardian as well as an assent from the CYP.

### Data collection

Data were collected between April and July 2015. Stakeholder engagement workshops were implemented to identify statement criterion to support development of potential PCOM domains informed directly by stakeholders’ views and perceptions of experiences of inpatient care for CYP with self-harm/eating disorders. Stakeholder engagement workshops offer an extensive inclusivity approach in establishing what those who are immediately affected by services want from their care (Sequeira and Warner, 2007; Jagpal et al., 2020). In addition, statement criterion was drawn from published research. We used creative and arts-based approaches as well as a Nominal Group Technique (NGT) to capture stakeholders’ experiences and views as detailed below.

### Creative and arts-based approaches

Creative and arts-based approaches were used in the workshops as they are useful and robust methods of collecting feedback, especially with CYP (Water et al., 2020; Coad, 2020). Users of arts-based methods can express thoughts and experiences that may prove too difficult to put into words. Using the visual story telling CYP stakeholders were invited to contribute to a timeline focusing on their hospital admission and pathway of care (Sheridan et al., 2011). Subsequently, they reflected on their journey by commenting on their experiences and how they were feeling across the timeline using drawings, words or phrases. They also identified where and when they wanted their outcomes to be assessed.

Stakeholders’ reflective evaluation on either receipt or delivery of care was focused on three themes identified from published research; care delivery; environment and communication. A fourth category, ‘anything else’ was created in order to capture information that did not align to the aforementioned categories. Thoughts, feelings, events and experiences were documented as statements and compiled under the relevant category heading.

### Nominal Group Technique (NGT)

Nominal Group Technique is a structured form of group decision-making (Harvey and Holmes, 2012) which allows generation of original ideas to be developed within a group whilst ensuring involvement from everyone (Lloyd et al., 1999). It involves the ranking of ideas, statements or domains by stakeholders according to their importance to achieve consensus (Jones and Hunter, 1995; Tague, 2004). NGT was applied to both stakeholder generated statements, developed from the first creative/art-based activity, and literature generated statements, developed from the rapid review in order to establish consensus from the stakeholder group.

### Stakeholder generated statements

As part of the creative activity exercise, individual stakeholders wrote down general comments before identifying their ‘most important’ statement. Discussion was paused until everyone had completed the exercise for each of the three categories, consequently resulting in ideas being shared from all stakeholders (Horton, 1980). Subsequently, stakeholders were asked to identify their most important statement for each category (care delivery, environment and communication). Each individual ranked level of importance from 5 (most important) to 1 (least important). All stakeholders had an equal opportunity to ‘vote’ on what they perceive to be most important to them. In circumstances where there were fewer than five statements, statements were scored from most to least important.

### Evidence generated statements

For all stakeholder events, each participant was given a booklet of evidence with statements generated from published research (Supplementary file). Like the stakeholder generated statements these were categorised into ‘care’, ‘communication’ and ‘environment’. Stakeholders were then asked to rank the top five statements according to how much they reflected their experience and outcome following receipt or delivery of care (5 – most important to 1 – least important).

### Engagement workshops

Individual workshops were conducted for each stakeholder group. All workshops had four consecutive activity structures (Supplementary file). For sensitivity purposes CYP workshops were divided into two groups based on their reason for admission: (i) those admitted due to an act of self-harm, and (ii) those who have been admitted due to an eating disorder. Two separate evening workshops were held for these groups to allow CYPs in full-time education an opportunity to attend. The events took place away from the hospital in a non-clinical environment. The team facilitating the events included health professionals with expertise in caring for CYP with mental health conditions to provide specialist advice and support if required.

### Data analysis

Data were collated and entered into Microsoft Excel 2013 (Microsoft Corporation). Data on experiences of receiving care were analysed using a data driven, iterative, thematic analytical approach (Braun and Clarke, 2006). Three researchers reviewed the raw data individually using spreadsheets. They then constructed themes through coding, combining, comparing and discussion (Cornish et al., 2013). Potential PCOM domain statements were ranked according to significance score calculated using the following equation: (score achieved for the item)/(maximum possible score) x 100, and allowed for the relative importance of the statements to be identified against the other statements (Gastelurrutia et al., 2009).

### Findings

A total of 96 stakeholders, 15 (16%) CYP, 8 (8%) parents and carers and 73 (77%) professionals took part in the study. Seventy-four CYP who had previously been admitted to acute paediatric care were identified from a local database by their treating clinician and contacted by letter inviting to participate. Fifteen (20%) of those eligible CYP agreed to participate. Of these, 11 were admitted following an episode of self-harm, while the other four were admitted with eating disorders. Eight CYPs attended the stakeholder events and the remaining participated by providing feedback over the telephone. Sixty-three parents and carers were contacted, with eight (13%) agreeing to take part in the study. The group comprised of parents and carers of children that had been admitted to hospital with either self-harm, eating disorders, or both. Non-attenders were invited via telephone or email to contribute to the study, but no additional feedback was collected. Of the 73 professional stakeholders, 36 (49%) attended stakeholder events with the remaining 37 (51%) providing feedback either face to face, via email or over the telephone.

### CYPs experiences of care

Clear variations in care pathways and experiences were reported between those admitted with self-harm and those admitted with eating disorders. Stakeholders experienced multiple inpatient transitions following initial presentation to inpatient care and delays to care as supported by statements below (please note pseudonyms have been used to protect anonymity of stakeholder):

‘Got moved ward three times’ (James, CYP with self-harm)

‘CAMHS took 2 days to come’ (Nyra, CYP with self-harm)

‘My mum spoke to the lady behind the desk and we were told to wait and we did for two hours’ (Salina, CYP with self-harm)

‘Lots of waiting for various doctors, appointments and meetings – nerve wrecking’ (Helen, CYP with eating disorder)

CYP alluded to the inappropriateness of certain inpatient areas for the receipt of care with examples of recognising the lack of age-appropriate environments or being exposed to additional traumatic experiences and events.

‘Babies and toddlers were loud and it would've been better to be with my age group’ (Billy, CYP with eating disorder)

‘There was different cases mixed it would have been better if it was people in the same situation’… ‘The cases around me weren’t appropriate, I watched a man die’ (James, CYP with self-harm)

‘When I was put on the cancer ward it made me wish I could switch places with the little boy opposite of me as he did not have a choice whether he could live or die’ (Salina, CYP with self-harm)

‘I was in a ward with cancer patients which were children, it made me feel uncomfortable’ (Arvind, CYP with self-harm)

Positive experiences of pathways of care were also evident from the CYP timelines. These related to environment, positive interactions with staff, feeling safe and informed, and the opportunity to interact with friends and peers.

‘Young person friendly, e.g., colourful pictures, only didn't like not allowed to close curtains’… ‘I met a lot of nice people’ (Billy, CYP with self-harm)

‘There was always somebody to speak to if I felt unsafe, worried or panicked’…‘The three nurses that looked after me were really helpful and explained everything really well’ (Arvind, CYP with self-harm)

*‘Friends visited me – yay!’* (Rochelle, CYP with eating disorder)

In addition to experiences of care, stakeholders also provided information as to how and when it was acceptable to report their outcomes. This differed between the two groups in that the CYP with self-harm reported that they wanted outcomes measured via an electronic device (such as a tablet or mobile telephone) prior to discharge. Whereas the CYP with eating disorders identified that they wanted to discuss their outcome directly with health professionals at 6 months following discharge or when they felt they had recovered enough. Supplementary file details themes and codes identified from the CYP stakeholder feedback.

### Ranking of CYP generated statements

Following thematic analysis of the feedback, CYP generated statements or topics were ranked by the CYP according to their importance (Table 2). The top five statements with the greatest strength score for CYP admitted with self-harm injuries were aligned to hospital environment, communication, and choices/boundaries, similarly those admitted with an eating disorder related to aspects of care, communication and environment.

**Table 2.** Statement ranking totals and strength of vote score of CYP generated statements from CYP (*n* = 8) admitted with self -harm injuries (*n* = 5) and eating disorders (*n* = 3).

Reason for admission	Statements	Total	Strength of vote score, %
Self-harm	Was not allowed to close the curtain :( so had no privacy	22	88
	Nurses spoke to my mum about how I feel and what I was going through	23	92
	Whilst I was waiting I was left alone which was good	19	76
	Not allowed for a fag so I was kicking off and really angry	25	100
	There was no entertainment	20	80
Eating disorders	Not many carers understood eating disorders :(	15	100
	Good to have a more specific ward for people with eating disorders	15	100
	Drs + nurses were nice	14	93
	Transparent and explained things when asked	14	93
	Doctors did not know when they would visit- lots of waiting for appointments	12	80

### Stakeholders ranking of literature generated and workshop statements

Using statements evidence generated statements and stakeholders’ statements generated during workshops, all stakeholders ranked the statements in order of importance. The top five statements by each group were identified from the strength of vote score and is summarised in Table 3. The following five patient-centred outcome measure (PCOM) domains were identified from the data; 1) privacy and surveillance, 2) receiving holistic care, 3) making choices and being understood through timely, relevant and appropriate communication, 4) working together to plan and achieve care goals and 5) respect and empowerment. Across the two groups of CYPs, similarities existed for statements that had the highest strength of vote score. Both groups rated, *My care focused more on my physical health rather than how I felt* as most important, with other statements including, *I felt that I was pressured to talk* and *I felt that I didn’t have my own privacy* ranked in the top five.

**Table 3.** Comparison of stakeholder top ranking statements across groups.

Statements		CYP self-harm	CYP eating disorders	Parents/carers	Professionals (self-harm)	Professionals (eating disorders)	Domain
Environment	‘I felt that I didn’t have my own privacy'	X	X	X		X	**1**
	‘I felt that I was not understood’		X	X	X		**2**
	‘I felt like I was being watched all the time’		X		X	X	**1**
	‘I felt that people were judging me’				X	X	**5**
	‘I had to follow the rules’		X				**4**
	‘I did not like being with sick children’	X					**3**
Care	‘My care focused more on my physical health rather than how I felt’	X	X	X	X	X	**2**
	‘I was scared about what others thought of me’		X	X	X	X	**5**
	‘I did not feel that people who worked in the different parts of my care worked together’	X		X		X	**4**
	‘I felt that I had other problems that were not addressed’		X	X		X	**2**
	‘I didn’t know what was happening in my care’		X		X	X	**3**
Communication	‘I felt that I was pressured to talk’	X	X				**3**
	‘I needed to work with others to get better’		X				**3**
	‘I felt that I had no voice’		X				**4**
	‘I felt that I was being controlled’					X	**4**
Domain 1 – Privacy and surveillance; Domain 2 – Receiving holistic care; Domain 3 – Making choices and being understood through timely, relevant and appropriate communication; Domain 4 – Working together to plan and achieve care goals; Domain 5 – Respect and empowerment							

Differences between the groups were also evident. For CYP admitted with self-harm injuries they regarded the inappropriate environment for receipt of care, and lack of inter-professional working to deliver their care, as important topics that they could relate to.

In contrast, for CYP admitted with eating disorders, they attributed importance to statements that related to conforming to rules and being watched, not being understood, fear of being stigmatised, not being informed, and not having a voice. However, as evident from Table 2, more statements were included in the top five as the strength of vote score was the same for several rankings. This made it challenging to distinguish between statements.

It was evident that for parents and carers the most important statements related specifically to care and the environment in which care were delivered. Statements receiving the highest strength of vote score included, *My son/daughter felt that they were not understood* and *My son/daughter felt that they had other problems that were not addressed*. These indicate the importance of professionals understanding individual children or young people and working in a way that addresses their holistic needs. Furthermore, other statements ranked as important indicated that care delivery focuses on physical health and delivery is disjointed between professionals.

Despite being ranked separately and, in most instances, by different individuals from the professional groups, commonalities existed between the rankings of statements developed from literature. Statements including: *CYP felt that people were judging them*; *CYP were scared about what others thought of them*; and *CYP’s care focused more on their physical health rather than how they felt* received highest strength of vote score. These related to stigmatisation and the physical focus of care being delivered. Supplementary file shows comparative statement ranking totals and strength of vote score from all stakeholders.

## Discussion

Through engagement with CYP (and their carers) previously admitted to paediatric inpatient settings with primary presenting conditions of either self-harm injuries or eating disorders this study was successful in identifying appropriate and relevant domains for a PCOM that assesses quality and impact of non-specialised, acute paediatric care.

A synthesis of findings identified five domains that could be used to develop a PCOM in this patient population: 1) privacy and surveillance; 2) receiving holistic care; 3) making choices and being understood through timely; relevant and appropriate communication; 4) working together to plan and achieve care goals; and 5) respect and empowerment.

At face value our findings are key and relevant in enabling delivery of person-centred care, which is a multidimensional notion focusing on the individual person as opposed to reason for admission. As can be determined from proposed domains, PCOM stems from the need to promote dignity, respect, compassion (Leplege et al., 2007) enabling shared decision-making and CYPs engagement to inform care received (Harding et al., 2015). Consequently, all PCOM domains are contextually aligned to three categories; care, environment and communication in line with published literature on CYPs reported outcomes.

### Care

In view of their experiences during the admission journey, CYP reported that care received was not integrated. The acute inpatient admission phase primarily focused on medical stability, with secondary domains relating to emotional, psychological and social needs. This focus on physical rather than the psychological aspects of health and wellbeing was viewed as counterproductive to recovery. One reason for thus may be that during acute hospital presentation, caregivers find it easier in the short term to measure effects of medical treatments on physical determinants as compared to tackling underlying psychological issues.

Conforming to hospitalisation was a challenge for the CYP in this study. This was due in part to their presenting problem (self-harm, eating disorders) which required close monitoring during hospital stay so CYP felt their privacy and rights were restricted and they were always under surveillance. Some reported that they were ‘doing time’ and recognised that this required them to conform to the rules to be ‘released’. Feelings of disempowerment and control were identified with CYP who felt punished for eating habits resulting in loss of using the telephone, participating in activities and not being allowed to see family members. A recent systematic review of patient experiences of hospitalisation following self-harm injuries found similar experiences (MacDonald et al., 2020). However, within this study this view did not reflect all CYP views as for some, environment and structure provided support and stability, again this was similarly reported by MacDonald et al. (2020).

Uncertainties were identified from CYP reports in relation to the planning of care and goals of inpatient stay. More ‘community resource planning’ was requested whereby greater focus was required on discharge planning and making follow up arrangements. This highlighted a need for continued support for families to access community based services and help to avoid them feeling unsupported and isolated (Grealish et al., 2013).

Nonprofessional and professional support were seen as equally important, with CYP needing support from parents or carers and the need of understanding and empathy from healthcare professionals. Parental or carers’ involvement has previously been shown to influence patient-centred care. Some CYP felt more supported when parents and carers were involved (Iachini et al., 2015), and trusted them to make decisions for them during health crisis (Grealish et al., 2013).

### Communication

Findings from the suggested PCOM domains highlight an urgent need to improve information provision for this patient population group and their parents or carers. Lack of sharing relevant information regarding treatment decisions with CYP and their parents or carers is likely a barrier to person-centred care as it severely restricts the potential for meaningful dialogue and negotiation with clinician and patient. CYP reported a lack of access to relevant information relating to fundamental aspects of care such as managing meals, activity levels and goal planning. This was perceived to affect self-esteem and social confidence, with CYP recognising that being informed and involved in decision making is fundamental to their recovery. From previous studies, it has been shown that being listened to and having information available enable CYP to maintain control and actively collaborate with care givers thus enabling the development of positive therapeutic relationships and trust (Iachini et al., 2015; Hart et al., 2005).

In summary, communication must be tailored to the individual and information should be easily comprehensible and free of jargon, with any written material produced through age-appropriate patient and public involvement groups.

### Inpatient environment

CYP reported that the inpatient environment created a sense of removal of the outside world and immobilised real-world involvement and development. For some CYP, admissions and readmissions were viewed as opportunities to escape and a place of safety from the complexities and difficulties of the outside world. However, for others, the inpatient environment rendered CYP removed from normality and disabled their agency over their own functioning. This appeared to have been exacerbated by CYP being on mixed wards with children experiencing acute, chronic and palliative conditions as well as exposure to younger children. Whilst this has not previously been reported with this patient group in an acute paediatric care setting, similar themes have been identified from qualitative accounts of adolescents and adults admitted to acute mental health settings (Wood and Alsawy, 2016; Offord et al., 2006; Cully et al., 2020). Moreover, these papers support the notion of conflicting experiences and perceptions of inpatient care with the environment ranging from a ‘safe haven’ (Wood and Alsawy, 2016: p. 38; Cully et al., 2020: p. 6) to being ‘removed from normality’ (Offord et al., 2006: p. 379).

Peer relationships within the inpatient environment were viewed both positively and negatively. Being with ‘similar others’ was reported to facilitate support and understanding through the sharing of the same experiences. Positive experiences of peer support were associated with feelings of acceptance by others with a similar condition, which contrasted to the stigma experienced from the external world. Similar experiences have been described from patients in acute mental health settings, who identify peer relationships offered support, reducing feelings of isolation, and enhanced understanding, contributing to positive experiences (Cully et al., 2020; Wood and Alsawy, 2016). However, in this study, peer relationships were also reported to be detrimental due to competitive interactions, making negative comparisons to others, and imposed segregation. Similar to findings from the qualitative study by Wood and Alsawy (2016) which identified comparisons with others resulted in guilt, competition, and negative thoughts and feelings, we also identified that CYP learned and subsequently adopted negative coping strategies, such as self-harming behaviours.

### Limitations

Despite inviting many CYP and parent and carer stakeholders to participate in this study, only a limited number chose to be involved. Although comprehensive feedback was elicited from these stakeholders, it is not known whether their experiences were different from those that did not take part. Furthermore, this study was conducted with stakeholders from a specific geographical region within England. Therefore, the findings may have limited transferability to other geographical regions or health systems. Further, stakeholders involved in this study had divergent experiences as a result of being in receipt of impatient care for a multitude of reasons (severe eating disorders, self-harm, and suicidality) and as such likely prioritised different outcomes. Moreover, for some stakeholders a substantial amount of time had passed since their time within an inpatient paediatric setting which may have affected their assessment of priority outcomes to measure.

#### Implications for practice

Despite the aforementioned limitations, this study included a diverse sample of stakeholders whose accounts have provided insights into the varied experience and quality of acute paediatric care of CYP with self-harm and eating disorders. However, despite this diversity, consensus in priorities for care experience and outcome are evident that include: Privacy and surveillance; Receiving holistic care; Making choices and being understood through timely, Relevant and appropriate communication; Working together to plan and achieve care goals; and Respect and empowerment. Health professionals within the acute paediatric healthcare services need to be highly cognisant of these when framing, delivering and evaluating their care for this patient group.

## Conclusion

This is the first study in the UK to define the preliminary domains for a PCOM for this patient group and part of the care pathway. However, further research is now needed to develop and evaluate the items within the PCOM domains for CYP admitted to acute paediatric care with self-harm or eating disorders. Our evaluative study demonstrates the need for the development of a PCOM to assess and address individual stakeholders’ expectations. Although there appears consensus in many areas, there remains differing views as to when and how outcomes are measured between the two groups of presenting conditions. Thus, any subsequent PCOM developed to evaluate the outcome of these patient groups needs to have the flexibility in how and when it is implemented to satisfy these diverse needs.

## Supplemental Material

Supplemental Material – Acute paediatric inpatient care of children and young people admitted with self-harm or eating disorders: A single centre evaluation Supplemental Material for Acute paediatric inpatient care of children and young people admitted with self-harm or eating disorders: A single centre evaluation by Joseph C. Manning, Tim Carter, Iszara Blake, Verity Bingham, Joanne Cooper, Andrew Turner, Takawira Marufu, Damian Wood and Jane Coad in Journal of Child Health Care
