# Supplementary material for: Acute paediatric inpatient care of children and young people admitted with self-harm or eating disorders: A single centre evaluation
Source: J Child Health Care. 2022 Jun 15;28(2):302–15. doi: 10.1177/13674935221107962 (PMC11141094; doi:10.1177/13674935221107962)
Supplement: Supplemental Material – Acute paediatric inpatient care of children and young people admitted with self-harm or eating disorders: A single centre evaluation [file sj-pdf-1-chc-10.1177_13674935221107962.pdf]

Outcome statements and categories from published data

Domain	Statement
Care	" My care focused more on my physical health rather than how I felt"
	"I did not feel that people who worked in the different parts of my care worked together"
	" I felt that I had other problems that were not addressed"
	"I didn't really feel cared for"
	"I was scared about what others thought of me"
	"I didn't know what was happening in my care"
Communication	" I felt that I was being controlled"
	"I needed to work with others to get better"
	" I felt that I was involved in decision making about my care"
	"I felt that I had no voice"
	"I felt that I was pressured to talk"
	" I did not feel that I had the opportunity to talk"
	" I felt that talking wasn't as important than my physical needs"
	"I felt that I understood what was going on"
	"I was active in planning my care"
	" I did not understand because I had no information about what was going on"
	" I felt alone because nobody spoke to me"
Environment	" I had to follow the rules"
	"I felt alone"
	" I felt isolated because I could not see my brothers/ sisters/friends/ parents"
	"I felt like I was being watched all the time"
	"I felt that I didn't have my own privacy"
	"I did not like being with sick children"
	" I felt that I was not understood"
	"I felt that people were judging me"

Stakeholders Workshop Structure

Activity	Criteria	Description
**1**	Icebreaker, introductions and getting to know each other	Event facilitators began by explaining the purpose of the event. The icebreaker activity itself is called ‘spider’s web’. It involves each of the CYP and facilitators throwing a small ball of wool to another and in turn telling the group a fun fact about themselves. The goal is to encourage the CYP to converse with each other and get to know each other in a fun way. Collectively, ground rules were developed and documented to ensure all stakeholders were clear that they could share their experiences in a safe and confidential space.
**2**	Evaluating experiences	As described above, this activity involved the application of the creative methods to evaluate and explore experiences of being in receipt of care.
**3**	Domain evaluation	This activity involved the ranking of both participant generated domains/statements and those that had been identified from the rapid review of the evidence.
**4**	Debrief/Tree of affirmation	Due to the sensitive and potentially evocative nature of the topic being evaluated, a debrief was conducted with all stakeholders . This provided opportunity to:<br>a. acknowledge anything they have learned from the event;<br>b. disclose anything that has not been covered during the other activities;<br>c. reflect on their experience of being involved in the project<br>d. ask the facilitators any questions they may have.

Themes and codes identified from the CYP stakeholder feedback (ED= eating disorder; SH= Self-harm)

Themes	Codes	SH	ED
**Burden of repetition**	Burdened by questioning; Same questions- different people	✓	✓
**Good communicatio n**	Nurse as mediator; Inclusion of parents; Respectful staff/positive interactions; Feeling catered for; Privacy; Continuity of communication; Accessibility to therapeutic communication	✓	
**Sense of normality**	Distraction; Nurses developed a therapeutic communication		✓
**Inclusive communicatio n**	Family centred care/inclusion of parents; Clarity of expectations; Feeling understood		✓
**Uncertainty**	Uncertainty about treatment; Uninformed- lack of communication; Unsafe/uncomfortable	✓	
**Surveillance**	Lack of control; Lack of freedom; Feeling constrained	✓	✓
**Feeling less important**	Misunderstood; Complex case; Recognising difficult/unpopular patient; Observing disparities in care provided		✓
**Access to therapeutic communicatio n**	Person centred care; Negotiated care; Taking time to care; Appreciation of nursing care; Good care/friendly staff		✓
**Focus on physical care**	Physical health assessment; Physical care priority over mental health		✓

Statement ranking totals and strength of vote score from all stakeholders

Stakeholder group and sample size (n)	Statements	Total	Strength of vote score
CYP admitted with self-harm injuries (n=11)	" My care focused more on my physical health rather than how I felt"	33	60%
	"I felt that I didn't have my own privacy"	30	54%
	"I felt that I was pressured to talk"	27	49%
	"I did not like being with sick children"	25	45%equal fourth
	"I did not feel that people who worked in the different parts of my care worked together"	25	45%equal fourth
CYP admitted with eating disorders (n=4)	" I felt that I was not understood"	14	70% equal 1st
	" My care focused more on my physical health rather than how I felt"	14	70% equal 1st

	"I felt that I had no voice"	13	65%
	"I felt that I didn't have my own privacy"	10	50% equal 3rd
	" I felt that I had other problems that were not addressed	10	50% equal 3rd
	"I felt that I was pressured to talk"	10	50% equal 3rd
	" I had to follow the rules"	9	45% equal 4th
	"I felt like I was being watched all the time"	9	45% equal 4th
	"I was scared about what others thought of me"	9	45% equal 4th
	"I didn't know what was happening in my care"	9	45% equal 4th
	"I needed to work with others to get better"	9	45% equal 4th
Parents and Carers (n=8)	"My son/daughter felt that they were not understood"	29	73%
	"My son/daughter felt that they had other problems that were not addressed"	28	70%
	"My son/daughter felt that they didn't have their own privacy"	24	60% equal 3rd
	" My son/daughter's care focused more on my physical health rather than how they felt"	24	60% equal 3rd
	"My son/daughter did not feel that people who worked in the different parts of their care worked together"	23	58%
	"My son/daughter was scared about what others thought of them"	21	53%
Professionals in relation to self-harm (n=50)	" CYP's care focused more on their physical health rather than how they felt"	87	62% equal 1st
	"CYP were scared about what others thought of them"	87	62% equal 1st
	"CYP felt like they were being watched all the time"	83	59%
	"CYP felt that they didn't have their own privacy"	81	58%
	"CYP felt that people were judging them"	69	49% equal 4th
	"CYP did not feel that people who worked in the different parts of their care worked together"	68	49% equal 4th
	" CYP felt that they were being controlled"	68	49% equal 4th
	" CYP felt that they had other problems that were not addressed	57	41% equal 5th
	"CYP didn't know what was happening in their care"	58	41% equal 5th
Professionals in relation to	" CYP's care focused more on their physical health rather than how they felt"	87	62% equal 1st

Eating Disorders (n=28)	"CYP were scared about what others thought of them"	87	62% equal 1st
	"CYP felt like they were being watched all the time"	83	59%
	"CYP felt that they didn't have their own privacy"	81	58%
	"CYP felt that people were judging them"	69	49% equal 4th
	"CYP did not feel that people who worked in the different parts of their care worked together"	68	49% equal 4th
	" CYP felt that they were being controlled"	68	49% equal 4th
	" CYP felt that they had other problems that were not addressed	57	41% equal 5th
	"CYP didn't know what was happening in their care"	58	41% equal 5th
